# Association between Free Sugars Intake and Excessive Daytime Sleepiness among Chinese Adolescents

**DOI:** 10.3390/nu13113959

**Published:** 2021-11-05

**Authors:** Yue Xi, Qian Lin, Qiping Yang, Fang Li, Hanmei Liu, Jing Luo, Yufeng Ouyang, Minghui Sun, Cuiting Yong, Caihong Xiang, Wenya Zheng

**Affiliations:** 1 Department of Nutrition Science and Food Hygiene, Xiangya School of Public Health, Central South University, 110 Xiangya Road, Changsha 410078, China; xiyue0404@csu.edu.cn (Y.X.); linqian@csu.edu.cn (Q.L.); yangqiping12@csu.edu.cn (Q.Y.); hanmeiliu@csu.edu.cn (H.L.); luojing2546@csu.edu.cn (J.L.); oyyf0102@csu.edu.cn (Y.O.); sun.1234@csu.edu.cn (M.S.); yongcuiting@csu.edu.cn (C.Y.); xch0622@csu.edu.cn (C.X.); 2 Department of Epidemiology and Health Statistics, Xiangya School of Public Health, Central South University, Changsha 410078, China; lifang_csu@csu.edu.cn; 3 Hunan Provincial Key Laboratory of Clinical Epidemiology, Changsha 410078, China

**Keywords:** adolescent, dietary sugars, sugar-sweetened beverages, excessive daytime sleepiness, sleep hygiene

## Abstract

This study aimed to investigate the prevalence of excessive daytime sleepiness (EDS) and explore the association between free sugars intake and EDS. In this cross-sectional study, a total of 1517 middle school students (808 boys and 707 girls) aged 12~14 years were recruited. The study was conducted in Changsha city, China. Adolescents completed an online questionnaire, including the Epworth Sleepiness Scale (ESS), sleep characteristics, a 12-item Food Frequency Questionnaire (FFQ), and other self-reported information. The ESS score ≥ ten was defined as EDS. The anthropometric indices, including height, weight, and waist circumference, were measured and recorded by uniformly trained assistants. Statistical analyses included the Chi-square test and binary logistic regression model. The mean ESS score and free sugars consumption were 6.8 ± 3.9 points and 53.1 ± 44.7 g/d, respectively. The prevalence of EDS among adolescents was 22.5%, and more girls than boys had EDS (26.1% vs. 19.4%, *p* < 0.05). An exceeded free sugars intake was positively associated with EDS, with the adjusted Odds Ratio (OR) with its 95% Confident Interval (95% CI) of 1.366 (1.060~1.761, *p* < 0.05). EDS and excessive consumption of free sugars are commonly found among Chinese adolescents. Further studies are needed to confirm whether free sugars restriction can be meaningful to improve daytime drowsiness in those with EDS.

## 1. Introduction

Excessive daytime sleepiness (EDS) is characterized by difficulty maintaining wakefulness and an increased tendency to fall asleep during the day, even after a whole night’s sleep [1]. Excessive sleepiness during the daytime is a common phenomenon worldwide nowadays. The prevalence of EDS ranged from 2.5 to 15.3% in the general adults [2,3,4,5]. It is estimated that over 40% of adolescents suffer from EDS [6,7]. In China, around 10.3% of adolescents higher than the 7th grade struggle with EDS [8]. As a public health concern, EDS is associated with various adverse consequences, including poor academic performance, mental health, accidental injury, and even adult crime [9,10,11,12].

EDS is related to several factors, including short sleep duration, obesity, depression, and screen time, etc. [2,13,14,15,16,17]. Apart from the above-known associations, the relationship between dietary patterns, especially sugar intake, and EDS is increasingly recognized. C. Anderson and J.A. Horne have revealed that high sugar content and low caffeine drinks would not alleviate sleepiness but rather worsen it [18]. Katagiri R et al. [19] suggested that high carbohydrate meals, including energy drinks and sugar-sweetened beverages, were associated with self-reported poor-quality sleep. A study by St-Onge et al. [20] showed that high-sugar intake was associated with less restorative sleep and more arousal, leading to EDS. The relationship between sugary drinks and sleep has been studied, but our understanding remains insufficient. Sugar-sweetened beverages (SSBs) were the primary contributors of dietary sugars. Within dietary sugars, it is free sugars that proved to be pivotal in impairing health, particularly in developing chronic diseases [21]. The term free sugars, as used by the World Health Organization (WHO) in its report, encompassed “all mono and disaccharides added to food by manufacturer, cook or consumer plus those sugars naturally present in honey, syrups and fruits juices, and concentrates” [21]. WHO recommended that, in both adults and children, the free sugars intake should reduce to less than 10% of total energy intake [21]. One genuine concern, up to now, is the growing consumption of free sugars. Free sugars intake of adolescents in Latin American countries ranged from 58.2 to 106.9 g/d [22], and in China with 22.5 g/d in 2012 [23] to 53.1 g/d in 2019 [24]. Commonly, free sugars intake accounted for more than 10% of energy in the diets [25]. Despite the growing number of studies conducted on sugar intake and sleepiness, the evidence mainly focused on EDS, dietary carbohydrates, and dietary SSBs [24,25,26,27,28], and the research on both free sugars intake and free sugars consumption frequency with sleepiness is relatively scarce.

Adolescence is a particular period for adulthood development and lifelong health [29,30]. During puberty, significant changes appear in sleep patterns due to biological factors, environmental factors, and social demands, increasing this fragility for the emergence of inadequate sleep [31], which could directly lead to EDS. Along with perceived study stress, physical inactivity, and more sedentary behaviors, adolescents might consume sugary foods and SSBs [32] more frequently for refreshment, responding to stress, or alleviating depressive and anxiety emotions [33,34]. All these factors might impair control of overeating sweets [35], contributing to the increased consumption of sugars by adolescents worldwide [25]. Despite the increasing interest in understanding sleep patterns and diet, studies conducted on this age group are limited.

To our best knowledge, no study has yet examined free sugars intake and its association with EDS in adolescents. Given that puberty is suggested to be a “critical period” for developing various diseases in later life, and dietary habits have been shown to track into adulthood, it is vital to explore the relationship between EDS and free sugars intake during this specific period. Thus, this study aims to investigate the prevalence of EDS and its association with free sugars intake among 12~14-year-old adolescents in China. We hypothesized that the consumption of free sugars is positively associated with EDS.

## 2. Materials and Methods

### 2.1. Ethical Approval

The study was approved by the Ethics Review Committee of the Xiangya School of Public Health, Central South University (XYGW-2019-025).

### 2.2. Study Design and Participants

From March to July 2019, we conducted this study in Changsha, the capital city of Hunan Province in south-central China. Ten middle schools from five districts (Yuhua, Tianxin, Furong, Kaifu, and Yuelu District) were selected using two-stage stratified random cluster sampling methods. Two middle schools were randomly sampled from each district. According to the inclusion and exclusion criteria, the 7th- and 8th-grade students were recruited when: (1) schools agreed to participate with more than 500 students, and (2) parents or caregivers agreed to participate, and consented to the collection of information. The exclusion criteria was the inability to read or write the questionnaire.

The protocol was submitted to the local Education Bureau for permission to conduct this investigation. With the education bureau’s assistance, we contacted leaders of the selected schools and then delivered the consent forms to students as homework. If parents or caregivers signed informed consent, the students were enrolled. A total of 1517 students completed the questionnaire (Valid response rate = 100%).

### 2.3. Measures

The survey included an online questionnaire and anthropometric measurements.

#### 2.3.1. Questionnaire Survey

The students were guided to complete the online questionnaire by uniformly trained research assistants in the schools’ computer room. The Questionnaire Star, an online questionnaire tool, was used to develop and deliver the investigation questionnaire. Each questionnaire had a unique linkage. We collected students’ demographics information, free sugars consumption, excessive daytime sleepiness, sleep characteristics, physical activities, sedentary and screen time, and laryngopharyngeal reflux symptoms.

- Demographic information: Students’ sex, age, sibling status (the only child or not), domicile place, and family monthly income were collected.
- Free Sugars Consumption: Free sugars consumption was collected by a food frequency questionnaire (FFQ) focused on sugar-sweetened beverages and sweetened foods. The FFQ was modified based on the two previous studies conducted by the Center for Diseases Control and Prevention of China (China’s CDC) and the result of the pilot study [36]. In the current study, SSBs consisted of carbonated drinks (also named sodas), vegetable protein drinks, juice or juice drinks, tea or tea drinks, sports drinks, and bubble tea. Sugary foods included cakes, desserts, candy (chocolate, Snickers and Maltesers, etc.), and preserved fruits (dried or candied fruits). We also set honey and flavored milk/yogurt as sources of free sugars. The response options of the average intake of SSBs and sugary foods included “100 mL/time, 200 mL/time, 300 mL/time, 400 mL/time, and 500 mL/time” and “25 g/time, 50 g/time, 75 g/time, 100 g/time, 150 g/time, and 200 g/time”, respectively.
- Excessive Daytime Sleepiness: The Epworth Sleepiness Scale (ESS) [37,38] was used to assess EDS. The ESS contained eight items that measure the probability of doze in different situations, with four responses scored from “0” to “3”. The results of ESS ranged from 0 to 24, with high scores reflecting high levels of sleepiness. In this study, ESS ≥ 10 was defined as EDS [39].
- Sleep Characteristics: Self-reported sleep characteristics, including sleep duration on weekdays and weekends, were obtained by three self-designed questions, including (1) In the last month, how many hours did you sleep on weekdays on average during a 24-h period? (2) In the last month, how many hours did you sleep on weekends on average during a 24-h period? The response options of these two questions ranged from “less than six hours” to “more than eight hours.” (3) How would you describe your sleep quality in the last month? Students could choose their answers from five options, categorized from “very good” to “moderate” to “very bad.”
- Physical activities: The short form of the International Physical Activity Questionnaire (IPAQ-short form) [40] was used to assess physical activity. The overall index of metabolic equivalent (MET, min/week) was used to present the intensity of physical activity. According to the recommended categorical scoring, students’ physical activity levels were divided into three groups: low activity (inactivity), moderate activity, and high activity.
- Sedentary time and screen time: Sedentary time was collected from IPAQ-SF, by asking “In the last seven days, how many minutes of the working days did you sit?” The answers were converted from minutes into hours, and according to WHO [41], sitting time > 8 h was defined as sedentary. Similarly, screen time was assessed by asking, “In the last seven days, how many minutes of the working days (or weekends) were you in front of the screen?” We then converted the answers into hours and took the mean of the total hours of workdays and weekends screen time as the daily screen time. Screen time > 2 h per day was categorized into longer screen time according to WHO [41].
- Laryngopharyngeal Reflux Symptoms (LPR symptoms): Symptoms were also assessed using the reflux symptom index (RSI) [42]. The RSI consists of 9 items with a 6-point response scale (from “No problem = 0” to “Severe problem = 5”). RSI scores ranged from 0 to 45; the higher the RSI score, the more severe the LPR symptoms. In this study, the RSI greater than 13 was identified as abnormal and likely LPR symptoms [42].

#### 2.3.2. Anthropometric Measurements

Conducted by uniformly trained research assistants, the height was measured by the standard height meter and weight by TANITA human body composition analyzer BC-W02C (Guangdong Food and Drug Administration (prospective), No. 2210704, 2014). The waist circumference (WC) was measured by calibrated flexible non-stretch tape laid. Body mass index (BMI) was calculated by dividing body weight (kg) by height squared (m^2^). Participants were classified as the standard and overweight/obese group based on the age- and sex-specific BMI cut-off values for Chinese children and adolescents (6~18 years old) [43]. Based on the age- and sex-specific cut-off values [44], the WC was grouped into three as normal (<P_75_), normal-high (P_75_–P_90_), and high (>P_90_).

### 2.4. Free Sugars Intake Assessments

The primary sources of free sugars were derived from SSBs, and sweetened foods included in the 12-item FFQ mentioned above. The response options of FFQ were converted to daily consumption frequencies as follows: 0 = “once a month or less”, 0.14 times/day = “once a week”, 0.36 times/day = “2~3 times a week”, 0.64 times/day = “4~5 times a week” and 2 times/day = “2 times/day or more”.

The following formula (1) calculated the individual’s estimated daily intake of free sugars [24]:

(1) $$Z=A\left(f_{1}\right)\times \frac{A\left(i_{1}\right)}{7}\times c_{1}+B\left(f_{2}\right)\times \frac{B\;\left(i_{2}\right)}{7}\times c_{2}+\ldots +X\left(f_{n}\right)\times \frac{X\left(i_{n}\right)}{7}\times c_{n}$$

daily consumption of free sugars (*Z*, g/d); food types (*A*, *B*, ···, *X*); frequency (*f*, times/d); intake of each time (*i*, g, or mL); free sugars content from China CDC database (*c*, g/100 g or mL/100 mL; shown in Table A1), and *n* is a natural number. For certain foods absent from the database, the average sugar content of such foods was used as a substitution. According to the recommendation of the latest China’s dietary guidelines [45], students were divided into normal- and high-sugar consumption groups (50 g as the cut-off values).

### 2.5. Statistical Analyses

EpiData 3.0 software (The Epi Data Association, Odense, Denmark) was used for data entry, the IBM SPSS 24.0 software (IBM Corp., Armonk, NY, USA) was used for data analyses. Descriptive data were presented as a percentage or the mean with standard deviation. We used the Chi-squared test and non-parametric tests to analyze related variates for different groups. Binary logistic regression analysis was used to analyze the association between free sugars intake and EDS. The explanatory variables with *p* < 0.10 were included in the multivariate analyses by the binary logistic regression’s backward stepwise method. Significant levels were set at *p* < 0.05.

## 3. Results

### 3.1. Characteristics of Participants

There were 1517 students in 7th and 8th grade who participated in this cross-sectional study. Of these, 46.7% were girls, and 47.3% were 12 years old (Table 1). Overall, 55.3% were only children, and 92.2% were day-students. There were 27.3% of students who were overweight or obese, 21.7% with normal–high waist circumference, and 24.1% with high waist circumference. The average consumption of free sugars was 53.1 ± 44.7 g/d in the current study. Higher the grade, more the free sugars consumed (40.2% vs. 46.5%, *p* = 0.013). Compared with day-students, those who live in dormitories consumed more free sugars (42.3% vs. 54.2%, *p* = 0.012).

The mean ESS score was 6.79 ± 3.88. The prevalence of EDS among adolescents was 22.5% (Table 1). More girls than boys tended to be sleepy during the daytime (26.1% vs. 19.4%, *p* = 0.002). Students with normal–high waist circumference accounted for a higher proportion of EDS than those with normal ones (27.7% vs. 19.7%, *p* = 0.009).

### 3.2. Sleepiness Related Factors among 12~14 Years Adolescents by Free Sugars Intake

Based on the Dietary Guidelines for Chinese Residents (2016), it would be appropriate for adults to consume no more than 50 g of added sugars, and the preferable level of sugars intake was below 25 g/d. In the present study, 43.2% of the students consumed free sugars higher than the recommended max level (Table 2). Overall, 50.3% of the adolescents with EDS were high free sugar consumers, compared with 41.2% of those without EDS (*p* = 0.003). There was a significant tendency for shorter sleep duration with increasing free sugars intake (weekday sleep duration: *p*_-trend_ = 0.004, and weekend sleep duration: *p*_-trend_ = 0.005). The median score of the overall MET was 1375.0 min/week. More than one-fourth of the participants (29.3%) were physically inactive, and only 22.7% were physically active. Among these students, 8.1% had symptoms of laryngopharyngeal reflux. The free sugars intake of students with LPR symptoms was significantly higher than those who did not have LPR symptoms (56.1% vs. 42.1%, *p* = 0.003). The longer the adolescents’ screen time, the higher the free sugars intake (47.8% vs. 36.1%, *p* = 0.001). We did not observe the difference in self-reported sleep quality, physical activity, and sedentary time by free sugars intake level among this population. In addition, adolescents’ EDS varied significantly by sleep duration, self-reported sleep quality, and LPR symptoms (Table A3).

Free sugars intake was different in total ESS scores (Table A4). The ESS scores of the adolescents who overeat free sugars of 50 g/d were 7.20, almost one point higher than that of students who did not. Additionally, free sugars intake is also differential in the specific item of the Epworth Sleepiness Scale. When sitting and reading, sitting and talking to someone, sitting quietly after a lunch without alcohol, and in a car while stopped for a few minutes in the traffic, students who consumed more free sugars tended to obtain a higher ESS score.

### 3.3. The Association between Free Sugars Intake and EDS among Observed Adolescents

The binary logistic regression model was used to explore the association between free sugars intake and EDS (Table 3). Students who indulged in free sugars more than those with a relatively low intake were inclined to consider dozing during the daytime (OR = 1.366, 95% CI: 1.060~1.761, *p* < 0.05). Considering different sources of free sugars (Table A5), students who more frequently consumed SSBs (OR = 1.525, 95% CI: 1.160~2.005, *p* < 0.05), desserts (OR = 1.333, 95% CI: 1.017~1.748, *p* < 0.05), or confectioneries (OR = 1.613, 95% CI: 1.241~2.097, *p* < 0.05) found it easier to be sleepy during the daytime. Separating SSBs into parts, drinking vegetable protein beverages more than one time a week (OR = 1.460, 95% CI: 1.085~1.965, *p* < 0.05) seems to be a risk factor of EDS. The associations between vegetable protein beverage (OR = 1.439, 95% CI: 1.054~1.964, *p* < 0.05), confectioneries (OR = 1.548, 95% CI: 1.178~2.035, *p* < 0.05), and EDS remained after adjustment.

## 4. Discussion

The ESS scores and free sugars intake among 12~14-year-old Chinese adolescents were relatively high. It is worth noting that high consumption of free sugars was associated with a high score of EDS. Adolescents who are more senior, living in the dormitory during school years, having shorter sleep duration, and accompanied with LPR symptoms tend to consume more sugary foods and SSBs and hence might have a higher risk of EDS. Although the research related to free sugars intake and EDS is insufficient, this study suggests a need for sugar intake reduction and sleep improvement in middle school students, plus a possible link between the two.

In the current study, the prevalence of EDS was 22.5% among adolescents, lower than Brazilian students of 47.6% [46], California students with 25% [47], and Polish teens with 36.1% [12], but higher than that of the students in Korea with 15.9% in 2001 and 19.3% in 2014 [32,48]. This may be due to the different criteria for EDS (cut-off value as ten or eleven) [32,47,48], the physiological difference among races [46], local lifestyles [47], school shift [46], or to other factors. Compared with studies conducted in China, the prevalence of EDS is also lower than that of the students in Hong Kong of 29.7% at Tanner stage 3 [49], Guangdong students with 27.6% [8], and Shandong adolescent girls with 42.7% [50]. These discrepancies could be explained by different measure tools [8,49,50], course scheduling [11], and socio-cultural and economic characteristics [8] of each region of China. Consistent with the previous studies [6,12,46,50,51,52], our results verified that EDS is positively associated with females, insufficient sleep, and poor sleep quality. Taken together, these studies revealed that the issue of EDS is widespread among adolescents at home and abroad.

Our results presented that an exceeded free sugars intake is associated with short sleep duration, the influential factors of EDS. Similar findings suggested that poor dietary behaviors related to poor sleep were also observed in other countries [53,54]. A randomized crossover trial has demonstrated that sugar intake among adults led to significantly impaired sleep with more waking episodes and less restorative sleep [20]. Poor sleep would lead to EDS and deficient daytime functioning. As such, it is possible that adolescents who have an insufficient sleep seek out more sugars and caffeine to stay awake [54]. It is also argued that shorter sleep durations and EDS among adolescents might lead to an energy deficit, leading to higher consumption of energy/caloric dense foods in response [55]. Another explanation is that those sleepy adolescents may be less active due to fatigue and thus spend more time on sedentary activities such as mobile phones, which is also likely associated with the high consumption of sugary foods [54]. This could be reflected by our results that longer screen time is associated with higher free sugars intake. Even though the mechanisms relating short sleep duration to the consumption of sugars are not well understood, findings on this topic highlight the need for tailored education programs concerning good sleep hygiene and healthy eating habits in adolescents.

One significant finding of this study is the positive association between free sugars intake and EDS. Compared with the total dietary free sugars, earlier studies paid more attention to SSBs [54,55,56]. Consistently, our results showed that higher consumption of SSBs correlated with a higher risk of EDS [53,54,55,56]. Taking Chinese Confucianism tradition and intrinsic socio-cultural values, adolescents in China may be more vulnerable to study stress and peer pressure [57]. In order to stay up later to learn more, SSBs, especially caffeine beverages, might be more convenient drinks available to youths at midnight rather than fruits or vegetables [56]. Such access to SSBs might lead to EDS due to the stimulation properties of sugars and caffeine, and which, when consumed near bedtime, may affect adolescents’ sleep. Apart from SSBs, the primary sources of free sugars among Chinese adolescents were flavored milk/yogurt and confectionery [24]. In line with other research [19,54], confectionery is another risk factor of EDS. However, the association of flavored milk/yogurt with EDS was not observed in our investigation. Nevertheless, the relationship between free sugars intake and EDS may create a vicious cycle contributing to an unhealthy diet and chronic sleep issues [13,54,56].

As for the various types of SSBs, it is noteworthy that vegetable protein drinks (also named plant protein beverages) were a risk factor of EDS. Plant protein beverages are based on plants alternating to cow’s milk [58]. An increasing number of traditional plant-based beverages are known worldwide, such as soymilk, coconut milk, oat milk, etc. It is generally believed in China that nuts such as walnut and almond are beneficial to promote memory [59,60]. Additionally, compared with tea or bubble tea, known as rich in caffeine, the consumers tend to assume that plant protein beverages, usually named “milk”, are “healthy”. All these situations might contribute to a higher consumption frequency of plant beverages. However, the large discrepancy in nutrients of these products and their bioavailability compared to milk or water was discrepant. For instance, the protein content of non-fortified homemade almond drinks is much lower (about 85%) than cow’s milk, and sometimes syrups or sucrose might be added for flavor promotion [61]. A possible explanation for this association is the amino acid Tryptophan present in plant beverages, which stimulates sleepiness after the ingestion [62,63]. Another interpretation might finally come down to the impact of excessive free sugars content in these plant protein beverages on EDS. Unfortunately, studies specifically addressing the relation between plant drink consumption and sleep impact in children and adolescents are limited [58]. In light of the thriving acceptance and consumption of plant beverages, the impact of free sugars intake, especially plant drinks, on adolescents’ health should be of concern.

Along with the factors mentioned above, the coronavirus disease 2019 (COVID-19) pandemic would be another issue regarding adolescents’ consumption of sugars and EDS. Studies revealed longer sedentary and screen time and higher prevalence of sleep and emotional problems during the COVID-19 pandemic than ever before [64,65,66]; under this stress state, people tend to consume more energy-dense foods to soothe negative emotions [67,68,69,70], and a higher intake of sugary foods could lead to weight gain or obesity, as well as EDS. Amongst all groups, youths were particularly prone to lifestyle and dietary changes during the COVID-19 pandemic [71]. This bi-directional relationship might create another vicious cycle, affecting adolescents living habits and eating behaviors, tracking into later life stages, and contributing to their lifelong health [71]. Hence, though we did not investigate the free sugars consumption during the COVID-19 pandemic period, living in the post-pandemic era with the new normal characterized by “social distancing” and “shelter in place” [71], it is significant to put new emphasis on implementing healthy eating habits.

One notable finding in our study was that, though WC was associated with EDS in univariate analysis, we did not observe the association between obesity, WC, and EDS after adjustment. These findings seem contradictory with major related studies [72,73,74] but are similar to the previous [8,75,76]. There might be several plausible interpretations. First, in the relation between obesity and EDS, pubertal status is a vital covariate to consider. Puberty marks considerable changes in sleep and circadian patterns and sex differences regarding changes in body composition, metabolism, and endocrinology that could shape the influence of sleep on BMI [77] and may serve as a viable explanation for null findings in samples [74]. Second, it is suggested that traditional measures of abdominal obesity have been shown to correlate poorly with sleep disturbances [78]. Third, earlier studies regarding weight gain and EDS were mainly focused on the general adult population, while the present study was conducted in adolescents [8]. Finally, EDS is assumed to be the consequence of obstructive sleep apnea (OSA) [2], a sleep disorder characterized by repetitive episodes of upper airway obstruction, which appears to form a vicious cycle with obesity where each worsens another. The relation among obesity, EDS, and OSA is complex, which might sometimes be presented as a negative, or irrelevant association [2,75]. Though the mechanism of EDS remains unclear, obesity and free sugars intake among adolescents are the two challenging public health problems. Health professionals, parents, and teachers, playing pivotal roles in disseminating health messages, should be aware of the potential link between sugar reduction and sleep promotion as early possible.

This is the first study that focused on the association between free sugars intake and EDS. The major strength of the present study is the relatively large samples and standard quality control. Each participant’s weight, height, and WC were assessed and recorded by uniformly trained assistants; this guaranteed the reliability of the anthropometric information. Additionally, we used the Epworth Sleepiness Scale to determine daytime drowsiness, making results more comparable and credible. Moreover, under the supervision of the teacher and project assistants, participants had to complete the questionnaire before submitting it to ensure data integrity.

However, interpretation of the findings in our study should consider some limitations. First, due to the cross-sectional design of this study, the causal relationship between free sugars intake and EDS may not be identified. This study was conducted on adolescents in a capital city of central-south China; thus, the findings may not be extrapolated to adolescents in rural or other parts of China. Furthermore, the free sugars intake was measured using an FFQ, and some sources of free sugars in this FFQ were absent, such as caffeine beverages or sucrose in homemade dishes; this could not accurately reflect the actual consumption of free sugars. Furtherly, the FFQ we used did not distinguish the beverages that contained the free sugars from those that did not, which would overestimate the adolescents’ free sugars intake. Moreover, sleep characteristics, screen time, and sedentary time were assessed by a self-report questionnaire, which would lose some information. Sleep habits are a dynamic biological process regulated by the circadian system, and a single measure may not capture the full effects of sleep duration on EDS. Finally, admittedly, missing values may bias the results, despite the sensitivity analysis showing that the association between EDS and family monthly income (variables with missing data) were similar before and after imputation.

## 5. Conclusions

In this study, we found that both the prevalence of EDS and the consumption of free sugars were high. High consumption of free sugars was associated with a high score of EDS, and consuming SSBs and confectionery more frequently is associated with a higher risk of EDS among adolescents. Examining factors related to EDS in this vulnerable group is urgently needed. Improving the food environments in family and school and limiting the availability of SSBs, especially caffeine beverages and sugary foods, would be essential for addressing excessive free sugars consumption among adolescents and might alleviate students’ EDS. Further research should focus more on dietary patterns, eating habits, and their relationship with sleep problems. A high-quality prospective study including a large sample should be conducted to clarify the causal association between dietary sugars and EDS or other sleep problems.

## Figures and Tables

**Table 1:** Characteristics of adolescents by outcome variables (*n* = 1517, mean ± *SD*).

Variables	Total (*n* = 1517)	High Free Sugars Intake (656, 43.2%)	*p*	EDS (342, 22.5%)	*p*
Sex			0.130		0.002
Boy	808 (53.3)	364 (45.0)		157 (19.4)	
Girls	709 (46.7)	292 (41.2)		185 (26.1)	
Age			0.085		0.461
12	396 (26.1)	154 (38.9)		81 (20.5)	
13	717 (47.3)	314 (43.8)		170 (23.7)	
14	404 (26.6)	188 (46.5)		91 (22.5)	
Grade			0.013		0.117
7th	784 (51.7)	315 (40.2)		164 (20.9)	
8th	733 (48.3)	341 (46.5)		178 (24.3)	
Only child			0.393		0.470
Yes	839 (55.3)	285 (42.0)		195 (23.2)	
No	678 (44.7)	371 (44.2)		147 (21.7)	
Live in or out			0.012		0.582
Dormitory	118 (7.8)	64 (54.2)		313 (22.4)	
Home	1399 (92.2)	592 (42.3)		29 (24.6)	
Family Monthly Income #			0.042		0.065
Low	616 (40.6)	251 (40.7)		117 (19.5)	
Medium	542 (35.7)	230 (42.4)		139 (25.1)	
High	359 (23.7)	175 (48.7)		86 (23.6)	
BMI Status			0.243		0.081
Normal	1103 (72.7)	487 (44.2)		236 (21.4)	
OW/OB	414 (27.3)	169 (40.8)		106 (25.6)	
Waist circumference			0.100		0.009
Normal	822 (54.2)	376 (45.7)		162 (19.7)	
Normal–high	329 (21.7)	134 (40.7)		91 (27.7)	
High	366 (24.1)	146 (39.9)		89 (24.3)	

Compared by Chi-square test. BMI status: grouped by age- and sex-specific BMI cut-off values. High free sugars intake: free sugars intake >50 g/d. EDS: ESS score ≥ 10. OW/OB: overweight/obese. Family monthly income: low (<5000 RMB), middle (5000–9000 RMB), high (>9000 RMB). RMB: Renminbi, Chinese official coupons, 1 RMB ≈ 0.15 USD. # Data with missing values, the information presented in Table 1 is the imputation results. Sensitivity analysis was conducted for confirmation (Table A2).

**Table 2:** EDS Related Factors by the level of free sugars intake (*n* = 1517 %).

Variables	Total (*n* = 1517)	Free Sugars Intake		* p *
		≤50 g/d (861, 56.8%)	>50 g/d (656, 43.2%)	
Excessive Daytime Sleepiness (EDS)				0.003
EDS	342 (22.5)	170 (49.7)	172 (50.3)	
No EDS	1175 (77.5)	691 (58.8)	484 (41.2)	
Sleep Duration				
On Workdays				0.005
<6 h	145 (9.6)	65 (44.8)	80 (55.2)	
6~8 h	1061 (69.9)	607 (57.2)	454 (42.8)	
>8 h	311 (20.5)	189 (60.8)	122 (39.2)	
On Weekends				0.006
<6 h	84 (5.5)	35 (41.7)	49 (58.3)	
6~8 h	593 (39.1)	329 (55.5)	264 (44.5)	
>8 h	840 (55.4)	497 (59.2)	343 (40.8)	
Self-reported Sleep Quality				0.072
Low	284 (18.7)	144 (50.7)	140 (49.3)	
Moderate	497 (32.8)	291 (58.6)	206 (41.4)	
High	736 (48.5)	426 (57.9)	310 (42.1)	
Laryngopharyngeal Reflux Symptoms				0.003
No	1394 (91.9)	807 (57.9)	587 (42.1)	
Yes	123 (8.1)	54 (43.9)	69 (56.1)	
Physical Activities				0.176
Low	445 (29.3)	253 (56.9)	192 (43.1)	
Medium	728 (48.0)	427 (58.7)	301 (41.3)	
High	344 (22.7)	181 (52.6)	163 (47.4)	
Screen Time				< 0.001
≤2 h	595 (39.2)	380 (63.9)	215 (36.1)	
>2 h	922 (60.8)	481 (52.2)	441 (47.8)	
Sedentary Time				0.345
≤8 h	1138 (75.0)	638 (56.1)	500 (43.9)	
>8 h	379 (25.0)	223 (58.8)	156 (41.2)	

Compared by Chi-square test. EDS: ESS score ≥ 10. No EDS: ESS score < 10. LPR symptoms: RSI ≥ 13.

**Table 3:** Binary Logistic regression model of association between free sugars intake and EDS among adolescents (No EDS as the reference, *n* = 1517).

Variables	Crude OR (95% CI)	Adjusted OR (95% CI)
Free Sugars Intake (≤50 g = ref)	1.444 (1.134, 1.839) **	1.366 (1.060, 1.761) *
Sleep Duration (<6 h = ref)		
On workdays	0.641 (0.510, 0.806) ***	0.880 (0.676, 1.145)
On weekends	0.836 (0.687, 1.018)	0.992 (0.798, 1.232)
Night Sleep Quality (Poor = ref)		
Neutral	0.517 (0.375, 0.712) ***	0.573 (0.411, 0.800) **
Good	0.363 (0.267, 0.494) ***	0.431 (0.308, 0.603) ***
LPR symptoms (No = ref)	3.619 (2.482, 5.277) ***	3.002 (2.026, 4.446) ***

*** *p* < 0.001, ** *p* < 0.01, and * *p* < 0.05. EDS: ESS score ≥ 10. No EDS: ESS score < 10. Adjusted by sex, age, BMI status, and waist circumference, and screen time. LPR symptoms: RSI ≥ 13.

## Data Availability

The data that support the findings of this study are not publicly available due to the data containing information that could compromise participant privacy but are available from the corresponding author on reasonable request.

## Appendix A

**Table A1:** Food item, weight, and free sugars from China CDC.

Food Descriptor	Quantity	Types	Free Sugars (g)
Sodas	100 mL	9	9.79
Bubble tea	100 mL	7	7.89
Tea drinks	100 mL	6	4.31
Fruit or natural juice	100 mL	21	10.47
Vegetable protein beverage	100 mL	19	7.14
Sports drink	100 mL	6	8.22
Flavored milk/yogurt	100 mL	29	12.28
Biscuits, cakes	100 g	118	17.50
Chocolate, confectionery	100 g	14	44.63
Fruit preserves	100 g	12	55.04
Honey	100 g	2	73.00

**Table A2:** Sensitivity analysis of the association between characteristics and EDS of the adolescents (n%).

Variables	Original		*p*	Imputed		*p*
	No EDS	EDS		No EDS	EDS	
Family Monthly Income ^a^			0.193			0.065
Low	328 (80.4)	80 (19.6)		482 (80.5)	117 (19.5)	
Medium	280 (75.7)	90 (24.3)		414 (74.9)	139 (25.1)	
High	187 (75.4)	61 (24.6)		279 (76.4)	86 (23.6)	

^a^ Family income level missing of 491 cases. Compared by Chi-square test. EDS: ESS score ≥ 10. No EDS: ESS score < 10. Family monthly income: low (<5000 RMB), middle (5000–9000 RMB), high (>9000 RMB). RMB: Renminbi, Chinese official coupons, 1 RMB ≈ 0.15 USD.

**Table A3:** Correlates of Sleep by level of EDS (*n* = 1517, %).

Variables	Total (*n* = 1517)	No EDS 1175 (77.5%)	EDS 342 (22.5%)	* p *
Daily Sleep Duration				
On Workdays				0.001
<6 h	145 (9.6)	99 (68.3)	46 (31.7)	
6~8 h	1061 (69.9)	815 (76.8)	246 (23.2)	
>8 h	311 (20.5)	261 (83.9)	50 (16.1)	
On Weekends				0.048
<6 h	84 (5.5)	56 (66.7)	28 (33.3)	
6~8 h	593 (39.1)	460 (77.6)	133 (22.4)	
>8 h	840 (55.4)	659 (78.5)	181 (21.5)	
Self-reported Sleep Quality				<0.001
Low	284 (18.7)	181 (63.7)	103 (36.3)	
Moderate	497 (32.8)	384 (77.3)	113 (22.7)	
High	736 (48.5)	610 (82.9)	126 (17.1)	
LPR Symptoms				<0.001
No	1394 (91.9)	1111 (79.7)	283 (20.3)	
Yes	123 (8.1)	64 (52.0)	59 (48.0)	
Physical Activity				0.716
Low	445 (29.3)	343 (77.1)	102 (22.9)	
Medium	728 (48.0)	560 (76.9)	168 (23.1)	
High	344 (22.7)	272 (79.1)	72 (20.9)	
Screen Time				0.250
≤2 h	595 (39.2)	470 (79.0)	125 (21.0)	
>2 h	922 (60.8)	705 (76.5)	217 (23.5)	
Sedentary Time				0.838
≤8 h	1138 (75.0)	880 (77.3)	258 (22.7)	
>8 h	379 (25.0)	295 (77.8)	84 (22.2)	

Compared by Chi-square test. EDS: ESS score ≥ 10. No EDS: ESS score < 10. LPR symptoms: the RSI ≥ 13.

**Table A4:** The scores of each Epworth Sleepiness Scale item by the level of free sugars intake (*n* = 1517, mean ± SD).

Variables	Total (*n* = 1517)	Intake of Free Sugars		* p *
		≤50 g/d (*n* = 861)	>50 g/d (*n* = 656)	
Sitting and reading	1.06 ± 0.84	0.96 ± 0.80	1.20 ± 0.77	< 0.001
Watching TV	0.59 ± 0.84	0.57 ± 0.82	0.62 ± 0.87	0.417
Sitting, inactive in a public place (e.g., a theater or a meeting)	0.18 ± 0.51	0.19 ± 0.50	0.18 ± 0.51	0.698
As a passenger in a car for an hour without a break	1.59 ± 1.08	1.58 ± 1.08	1.60 ± 1.09	0.649
Lying down to rest in the afternoon when circumstances permit	1.85 ± 1.11	1.82 ± 1.12	1.89 ± 1.09	0.226
Sitting and talking to someone	0.16 ± 0.47	0.12 ± 0.39	0.22 ± 0.54	< 0.001
Sitting quietly after a lunch without alcohol	0.68 ± 0.89	0.63 ± 0.87	0.75 ± 0.91	0.006
In a car, while stopped for a few minutes in the traffic	0.67 ± 0.95	0.62 ± 0.91	0.74 ± 0.98	0.009
Total ESS Scores	6.79 ± 3.88	6.48 ± 3.79	7.20 ± 3.96	< 0.001

Compared by Kruskal Wallis test. ESS: Epworth Sleepiness Scale.

**Table A5:** Binary logistic regression models on the associations between EDS and consumption frequencies of different free sugars sources (No EDS as the reference, *n* = 1517).

Variables (≤1 Time/Week = ref)	Crude OR (95% CI)	Adjusted OR (95% CI)
SSBs #	1.525 (1.160, 2.005) **	1.509 (1.141, 1.996) **
Sodas	1.296 (1.000, 1.679)	1.244 (0.949, 1.630)
Bubble tea	1.424 (1.081, 1.876) *	1.297 (0.974, 1.726)
Tea drinks	1.269 (0.940, 1.714)	1.334 (0.974, 1.827)
Fruit or natural juice	0.990 (0.693, 1.413)	0.994 (0.686, 1.441)
Vegetable protein beverage	1.460 (1.085, 1.965) *	1.439 (1.054, 1.964) *
Sports drink	0.975 (0.633, 1.500)	0.948 (0.602, 1.494)
Flavored milk/yogurt	1.184 (0.927, 1.513)	1.199 (0.930, 1.545)
Cakes and Desserts	1.333 (1.017, 1.748) *	1.312 (0.990, 1.740)
Confectionery	1.613 (1.241, 2.097) ***	1.548 (1.178, 2.035) **
Fruit preserves	1.018 (0.705, 1.471)	0.974 (0.665, 1.425)
Honey	1.432 (0.932, 2.201)	1.398 (0.890, 2.196)

#: ≤ 4 times/week = ref. *** *p* < 0.001, ** *p* < 0.01, and * *p* < 0.05. EDS: the ESS score ≥ 10. No EDS: the ESS score < 10. Adjusted by students’ age, sex, BMI status, sleep duration, and self-reported sleep quality.
